# IL-23 Promotes Neutrophil Extracellular Trap Formation and Bacterial Clearance in a Mouse Model of Alcohol and Burn Injury

**DOI:** 10.4049/immunohorizons.2100109

**Published:** 2022-01-20

**Authors:** Xiaoling Li, Marisa E. Luck, Caroline J. Herrnreiter, Abigail R. Cannon, Mashkoor A. Choudhry

**Affiliations:** * Alcohol Research Program, Stritch School of Medicine, Loyola University Chicago Health Sciences Division, Maywood, IL; † Burn and Shock Trauma Research Institute, Stritch School of Medicine, Loyola University Chicago Health Sciences Division, Maywood, IL; ‡ Department of Surgery, Stritch School of Medicine, Loyola University Chicago Health Sciences Division, Maywood, IL; § Integrative Cell Biology Program, Stritch School of Medicine, Loyola University Chicago Health Sciences Division, Maywood, IL; ¶ Biochemistry and Molecular Biology Program, Stritch School of Medicine, Loyola University Chicago Health Sciences Division, Maywood, IL; || Department of Microbiology and Immunology, Stritch School of Medicine, Loyola University Chicago Health Sciences Division, Maywood, IL

## Abstract

Our previous studies have shown that ethanol intoxication combined with burn injury increases intestinal bacterial growth, disrupts the intestinal barrier, and enhances bacterial translocation. Additionally, studies show that Th17 effector cytokines IL-17 and IL-22, which are dependent on IL-23, play important roles in maintaining intestine mucosal barrier integrity. Recent findings suggest neutrophils are a significant source of IL-17 and IL-22. We determined the effect of ethanol and burn injury on neutrophil IL-17 and IL-22 production, as well as their ability to phagocytose and in bacterial clearance, and whether these effects are modulated by IL-23. Mice were given ethanol 4 h prior to receiving ~12.5% total body surface area burn and were euthanized day 1 after injury. We observed that intoxication combined with burn injury significantly decreases blood neutrophil phagocytosis and bacteria killing, as well as their ability to produce IL-17 and IL-22, compared with sham vehicle mice. The treatment of neutrophils with rIL-23 significantly increases IL-22 and IL-17 release and promotes expression of IL-23R, retinoic acid–related orphan receptor γt, Lipocalin2, and Nod-like receptor 2 following ethanol and burn injury. Furthermore, IL-22– and IL-17–producing neutrophils have enhanced neutrophil extracellular trap formation and bacterial killing ability, which are dependent on IL-23. Finally, although we observed that peritoneal neutrophils harvested after casein treatment are functionally different from blood neutrophils, both blood and peritoneal neutrophils exhibited the same response to rIL-23 treatment. Together these findings suggest that IL-23 promotes neutrophil IL-22 and IL-17 production and their ability to kill bacteria following ethanol and burn injury.

## INTRODUCTION

People drink alcohol (ethanol) to socialize, celebrate, and relax. However, drinking in excess, on a single occasion or over longer periods can lead to accidents and alcohol dependence. Alcohol abuse also suppresses the immune system; causes inflammation in the liver, brain, pancreas, gastrointestinal tract, etc.; and damages these organs [(Refs. 1—6); https://www.niaaa.nih.gov/publications/brochures-and-fact-sheets/hangovers]. In the United States, ~486,000 burn injuries receive medical treatment, and ~40,000 burn injuries require hospitalization every year (https://ameriburn.org/who-we-are/media/burn-incidence-fact-sheet/). Nearly half of burn injury patients have detectable positive blood alcohol concentration at the time of hospital admission. Studies have shown that patients under the influence of alcohol at the time of injury have increased risk for infection and higher morbidity and mortality than a similar burn injury without alcohol intoxication (1–3). Bacterial infections secondary to burn injury can lead to multiple organ dysfunction and failure and are major causes of increased morbidity and mortality in these patients. Both clinical and experimental findings indicate that alcohol intoxication combined with burn injury suppress immune function (3–6). Studies from our laboratory have indicated that alcohol intoxication combined with burn injury suppressed Th1 and Th17 effector responses, which are accompanied by increased intestinal bacterial overgrowth and translocation (7–10).

Neutrophils are the most abundant leukocytes of all innate effector cells and the first line of defense against many pathogenic organisms, including bacteria, fungi, protozoa, and viruses. During an infection, neutrophils quickly migrate through the endothelium of blood vessels to inflammatory sites. These activated neutrophils recognize pathogen-associated molecular patterns and eliminate pathogens through two mechanisms: phagocytosis, in which pathogens are engulfed and digested by proteases and reactive oxygen species (ROS) in phagolysosomes; and degranulation of granules containing antimicrobial proteins and peptides, including myeloperoxidase (MPO), neutrophil elastase (NE), cathepsins, and defensins (11–13). Neutrophils also release web-like structures composed of DNA decorated with histones and highly concentrated antimicrobial proteins, known as neutrophil extracellular traps (NETs). NETs are able to trap and kill extracellular pathogens (11–13).

Studies have shown that IL-22 and IL-17 play important roles in maintaining intestinal mucosal barrier integrity during infection by various pathogens, such as *Citrobacter rodentium* and *Staphylococcus aureus* (14, 15). IL-22 increases the presence of antimicrobial proteins within the mucosa, such as Lipocalin2, Reg3β, and Reg3γ, to clear pathogens and enhance epithelial cell proliferation and mucous production (9, 16–18). IL-17 and IL-22 can be produced by numerous lymphocytes, including Th17, Th22, innate lymphoid cells, NK cells, and γd T cells (8, 19–21). Recent studies have indicated that neutrophils are an important source of IL-22 and IL-17 (22, 23). IL-22–producing neutrophils protect against intestinal damage in DSS-induced colitis. IL-22–deficient mice receiving neutrophils from wild-type mice led to protection against DSS-induced intestinal epithelial cell damage. Furthermore, IL-22–producing neutrophils up-regulated antimicrobial peptides expression by epithelial cells (24). Neutrophils producing IL-22 and IL-17 are dependent on IL-23 through regulation by the IL-23R, retinoic acid–related orphan receptor γt (RORγt), and aryl-hydrocarbon receptor (AHR). Furthermore, IL-23 induces neutrophil IL-22 and IL-17 release through the mTOR pathway (23, 24). In this study, we determined whether IL-23–dependent induction of IL-17 and IL-22 production by neutrophils contributes to neutrophil NET formation and bacterial killing following ethanol intoxication combined with burn injury. We observed that ethanol intoxication combined with burn injury suppressed blood neutrophil phagocytosis and bacterial killing ability, which was accompanied with decreased neutrophil release of IL-22 and IL-17. The treatment of neutrophils with IL-23 promoted neutrophil IL-22 and IL-17 production, neutrophil NET formation, and bacterial killing following ethanol and burn injury.

## MATERIALS AND METHODS

### Animals and reagents

Male C57/BL6 mice (22–25 g) were obtained from Charles River Laboratories (Wilmington, MA). IL-17 ELISA kit was obtained from R&D Systems (Minneapolis, MN). IL-22 ELISA kit was obtained from eBioscience (San Diego, CA). Casein was obtained from Sigma-Aldrich (St. Louis, MO). EasySep Mouse Neutrophil Enrichment kit was obtained from STEMCELL Technologies (Vancouver, BC, Canada). Fixable Viability Dye eFluor 506, PE IL-22, FITC IL-17, and Cell Stimulation Cocktail (plus protein transport inhibitors) were obtained from eBioscience (San Diego, CA). Primers to IL-23R, RORc, Lipocalin2, Nod-like receptor 2 (Nod2) and GAPDH, MirVana miRNA Isolation Kit, High Capacity cDNA Reverse Transcription Kit, TaqMan Fast Advanced Master Mix, and pHrodo Red *Escherichia coli* BioParticles conjugate for phagocytosis were obtained from Thermo Fisher Scientific (Hampton, NH). Anti-histone H3 Ab (Citrulline 2, 8, 17) and anti-FITC MPO were obtained from Abcam (Cambridge, MA).

### Mouse model of acute ethanol intoxication and burn injury

As described previously (8, 9), 22- to 25-g male C57/BL6 mice were randomly divided into two groups: sham vehicle and burn ethanol. In the burn ethanol group, mice were gavaged with 0.4 ml of 25% ethanol (~2.9 g/kg), and in the sham vehicle group, mice were gavaged with 0.4 ml of water. In the burn injury group, mice were administered with buprenorphine (Simbadol C III) at a dose of 1 mg/kg body weight s.c. 1 h before burn injury. Four hours after the gavage, both groups of mice were anesthetized with a mixture of ketamine and xylazine by i.p. injection and transferred into a template fabricated to expose ~12.5% of the total body surface area on the dorsal surface. For burn injury, mice were immersed in 85–90°C water bath, and sham mice were immersed in a ~37°C water bath for ~7 s. Mice were dried immediately and resuscitated with 1.0 ml physiological saline by i.p. injection. After recovery from anesthesia, mice were returned to their cages and allowed food and water ad libitum. All of the animal procedures were carried out in adherence to the National Institutes of Health *Guide for the Care and Use of Laboratory Animals* and were approved by the Loyola University Chicago Health Sciences Division Institutional Animal Care and Use Committee.

### Isolation of neutrophils from peripheral blood and peritoneal cavity

One day after injury, mice were euthanized, and blood was collected by cardiac puncture. RBCs were lysed using ACK Lysing Buffer (Life Technologies). WBCs were collected by centrifugation at 1500 rpm for 10 min at room temperature. Blood neutrophils were isolated using the EasySep Mouse Neutrophil Enrichment kit (STEMCELL Technologies) according to the manufacturer’s instructions. For isolation of neutrophils from the peritoneal cavity (25), mice were injected i.p. with 1 ml of 9% casein in physiological saline instead of resuscitation with physiological saline. The next morning, mice were injected i.p. with a second dose of casein. Three hours after the second injection, mice were euthanized and injected with 8 ml of harvest solution (PBS supplement with 2% FBS and 2 mM EDTA) into the peritoneal cavity. After gently massaging the abdomen, peritoneal cavity fluid was slowly withdrawn. Peritoneal exudate cells were collected by centrifugation at 1500 rpm for 10 min at room temperature and were washed two times with PBS. The cells were resuspended in 2 ml of PBS, 3 ml of Histopaque 1119 (Sigma Aldrich) was added into a 15-ml tube, and 3 ml of Histopaque 1077 (Sigma Aldrich) was gently added on top of the Histopaque 1119 layer. The cell suspension was carefully added over the upper gradient and centrifuged at 2000 rpm for 30 min at room temperature without using a brake. Neutrophils were collected from the interface of Histopaque 1077 and 1119 layers.

### Measurement of neutrophil phagocytosis

One day after injury, mice were euthanized, and blood was collected by cardiac puncture. RBCs were lysed. WBCs were washed with wash buffer (PBS supplement with 5% FBS) twice. A total of 1 × 10^5^ cells were incubated with 100 μl of pHrodo Red *E. coli* BioParticles conjugate for 1 h and washed two times with cold wash buffer according to the manufacturer’s instructions. The cells were incubated with Fixable Viability Dye eFluor 506 for 30 min on ice for dead cell staining and washed twice with cold wash buffer. The cells were stained with Ly6G and CD11b Abs for 30 min on ice and washed twice with cold wash buffer. Cells were resuspended in 0.3 ml wash buffer and analyzed for neutrophil phagocytosis by FACS Canto II (BD Biosciences) and FlowJo software (Tree Star).

### Bacterial killing assay

*E. coli* were grown to log phase by culturing overnight (i.e., ~16 h) prior to using for bacterial killing assay (26). Isolated neutrophils (3 × 10^5^) in 100 μl RPMI 1640 supplemented with 10% FBS were placed in each well of a 48-well plate. Then, 9 × 10^6^ CFUs *E. coli* in 100 μl medium was added to each well (1:30) and incubated at 37°C in 5% CO_2_ for 1 h. The cells were washed with RPMI 1640 supplemented with 10% FBS, 50 μg/ml gentamicin, 100 U/ml penicillin, and 100 μg/ml streptomycin for three times to remove and kill extracellular bacteria. Neutrophils were lysed with 100 μl H_2_O. Then, 10 μl of original lysate and 1:10 diluted lysate were placed on a tryptic soy agar plate and incubated at 37°C overnight. The bacterial colonies were counted and calculated for each sample.

### Measurement of neutrophil IL-17 and IL-22

Blood cells (1 × 10^6^) were stimulated with Cell Stimulation Cocktail (plus protein transport inhibitors; catalog no. 00-4975; Thermo Fisher Scientific), which contains PMA, Ionomycin, brefeldin A, and monensin in cell culture medium for 3 h. The cells were harvested, washed with ice-cold PBS, and incubated with Fixable Viability Dye (1 μl/ml; eBioscience) for 30 min for dead cell staining. CD16/32 Ab (catalog no. 14-0161-85; Thermo Fisher Scientific) was added to block Fc receptors. The cells were stained with Ly6G and CD11b Abs for 30 min and washed with FACS buffer (5% FCS in PBS) two times. Typically CD11b^+^ and Ly6G^+^ cells are considered as neutrophils (25). Intracellular staining (IL-17 and IL-22) was performed using BD Cytofix/Cytoperm Fixation/Permeabilization Kit according to the manufacturer’s instructions. IL-17– and IL-22–positive neutrophils were analyzed by FACS and FlowJo software.

For measurement of neutrophil IL-17 and IL-22 release, isolated neutrophils (2 × 10^5^ cells/well) were cultured in RPMI 1640 supplemented with 2 mM/l l-glutamine, 10 mM/L HEPES, 50 μg/ml gentamicin, 100 U/ml penicillin, 100 μg/ml streptomycin, and 10% FCS (complete RPMI 1640) in 96-well plates in the presence or absence of rIL-23 (10 ng/ml) at 37°C and 5% CO_2_ for 24 h. The supernatants were harvested to determine IL-17 and IL-22 levels using ELISA kits according to the manufacturer’s instructions.

### Quantitative RT-PCR

Isolated neutrophils were cultured in complete RPMI 1640 in 96-well plates in the presence or absence of rIL-23 (10 ng/ml) at 37°C and 5% CO_2_ for 24 h. Cells were collected for isolation of total RNA using mirVana miRNA Isolation Kit according to the manufacturer’s instructions. The total RNA concentration was determined using a NanoDrop spectrophotometer (Thermo Scientific). A total of 300 ng of total RNA was used for cDNA reverse transcription by using a High Capacity cDNA Reverse Transcription Kit according to the manufacturer’s instructions. Gene expression was analyzed using specific primers with TaqMan Fast Advanced Master Mix on an Applied Biosystems Real-Time RT-PCR System and normalized with GAPDH.

### Measurement of NETs

NETs were analyzed using a modified protocol (27). Briefly, isolated neutrophils were cultured in complete RPMI 1640 in the presence or absence of rIL-23 (10 ng/ml) at 37° C and 5% CO_2_ for 3 or 16 h, then fixed in 2% paraformaldehyde for 15 min and blocked in staining buffer (PBS with 2% BSA) for 30 min. The cells were stained with anti-histone H3 Ab (Citrulline 2, 8, 17; Abcam), PE-conjugated secondary Ab (Thermo Fisher Scientific), FITC-conjugated anti-MPO Ab (Abcam), and DAPI (Thermo Fisher Scientific). The NETs were analyzed by flow cytometry and FlowJo software. Meanwhile, neutrophils were cultured with rIL-23 (10 ng/ml) in chamber slides for 16 h. The neutrophils were fixed and stained with anti-histone H3 Ab, PE-conjugated secondary Ab, and FITC-conjugated anti-MPO Ab under the same condition. The slides were then incubated with ProLong Gold antifade mounting reagent containing DAPI (Thermo Fisher Scientific). The NET images were observed by EVOS FLC fluorescence microscopy (Thermo Fisher Scientific) with ×400 magnification.

### Statistical analysis

The data are presented as means ± SEM and were analyzed with one-way ANOVA with Tukey multiple comparisons test or unpaired *t* test (GraphPad Prism 8.0.1; GraphPad.com). A *p* value <0.05 was considered statistically significant.

## RESULTS

### Ethanol intoxication combined with burn injury decreases neutrophil phagocytosis

**FIGURE 1.**
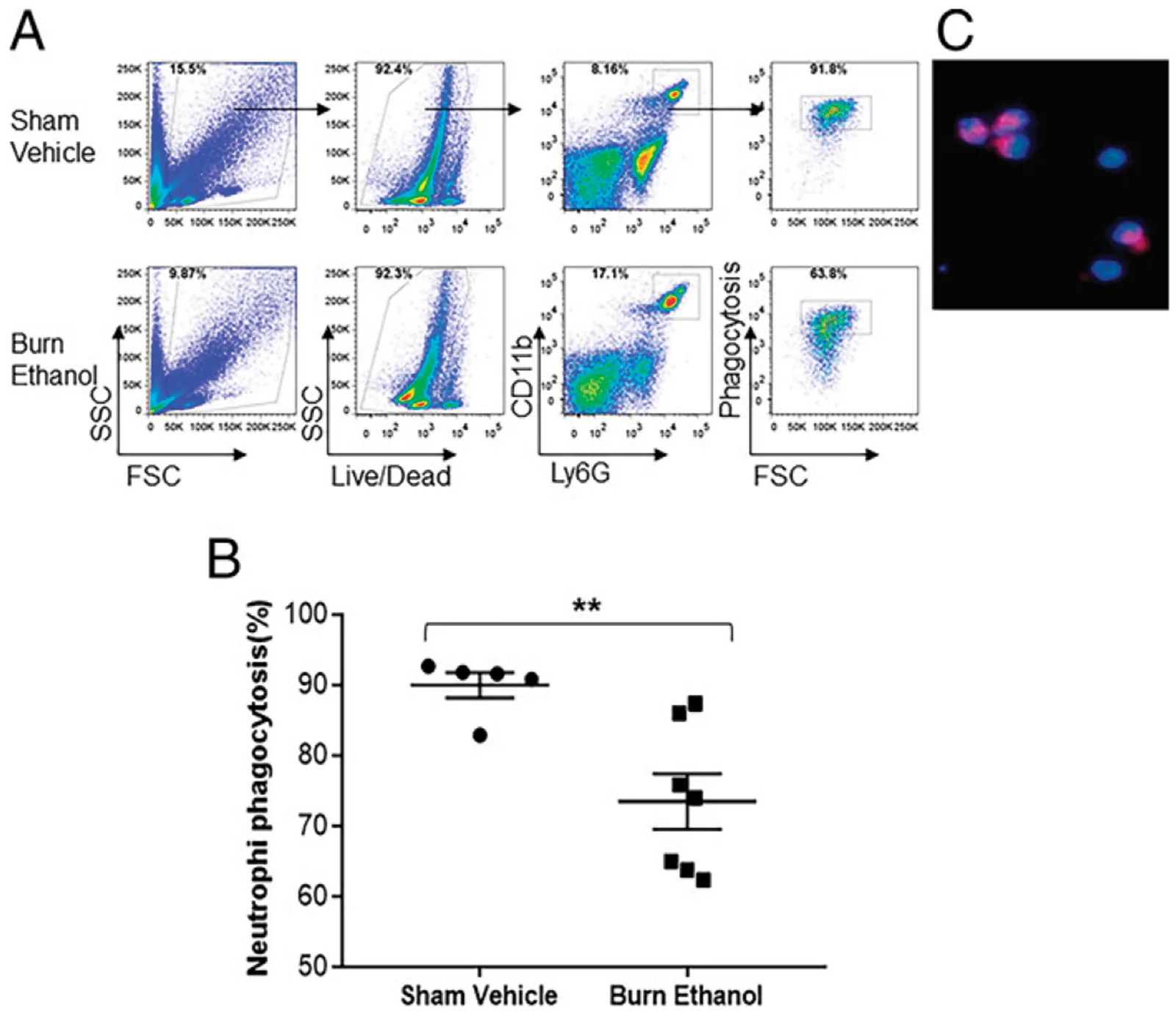
Decreases in neutrophil phagocytosis following ethanol intoxication combined with burn injury. One day after injury, the peripheral blood was collected, and RBCs were lysed. The total WBCs (3 × 10^5^ cells) were incubated with pHrodo Red–conjugated *E. coli* BioParticles for 1 h and then stained with Fixable Viability Dye, CD11b, and Ly6G Abs. We first used forward light scatter and side scatter (of light) to gate the cells. The dead cells were then gated out as observed by Fixable Viability Dye. Live cells with CD11b^+^, Ly6G^+^, and pHrodo Red^+^ were considered as neutrophil phagocytosis by FACS (**A**). Quantification of neutrophil phagocytosis (**B**). Photomicrograph of neutrophil phagocytosis (**C**). Values are means ± SEM from five to seven animals per group. **p < 0.01 compared with sham vehicle group by unpaired *t* test.

Neutrophils are the first line of defense against invading pathogens. Under inflammatory conditions, blood neutrophils quickly migrate from blood to sites of inflammation to kill pathogens by active phagocytosis or forming NETs. In a previous study from our laboratory, we observed that ethanol intoxication combined with burn injury increased bacterial translocation from the intestinal lumen. In this experiment, we determined whether ethanol intoxication combined with burn injury influenced neutrophil phagocytosis. To test this, 1 d after injury, blood was collected and RBCs were lysed. Total WBCs (1 × 10^5^ cells) were cultured with pHrodo Red–conjugated *E. coli* BioParticles for 1 h and then stained for Ly6G and CD11b to gate for blood neutrophils. As shown in Fig. 1, there was a significant decrease in neutrophil phagocytosis (73.50 ± 3.92%) in ethanol burn mice compared with sham vehicle mice (89.96 ± 1.79%).

### Ethanol intoxication combined with burn injury decreases the bacterial killing ability of neutrophils

**FIGURE 2.**
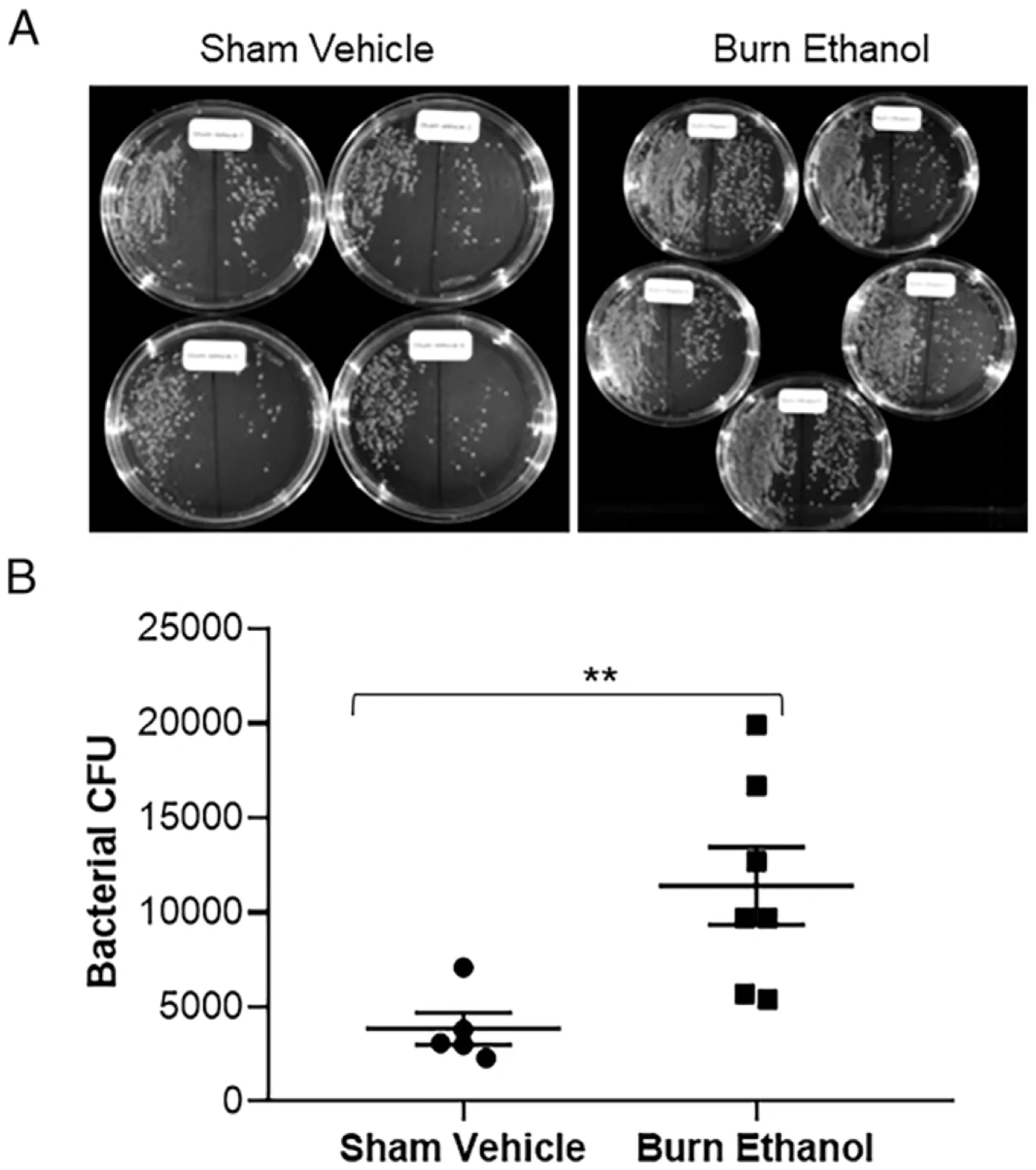
Decreases in neutrophil ability of bacteria killing following ethanol intoxication combined with burn injury. One day after injury, the peripheral blood neutrophils were isolated. Neutrophils (3 × 10^5^) were incubated with 9 × 10^6^ CFUs *E. coli* (1:30) for 1 h. The extracellular bacteria were killed by using gentamicin, penicillin, and streptomycin. The neutrophils were lysed with H_2_O. The 10 μl of cell lysis was placed on the tryptic soy agar plate and incubated at 37°C overnight. The bacteria colonies were counted and calculated for each sample. Each plate contained two concentrations of cell lysate. The left half of the plate shows undiluted cell lysates, and the right half demonstrates a 10× dilution of cell lysate (**A**). Values are means ± SEM from five to seven animals per group (**B**). **p < 0.01 compared with sham vehicle group by unpaired *t* test.

One day after injury, blood neutrophils were isolated using EasySep Mouse Neutrophil Enrichment kit (STEMCELL Technologies). Neutrophils were cocultured with *E. coli* (1:30) for 1 h. The extracellular bacteria were washed and killed with media containing gentamicin, penicillin, and streptomycin. Neutrophils were lysed, and intracellular bacteria were cultured and counted (left half of the plate is original lysate and right half is 1:10 diluted lysate). As shown in Fig. 2, we observed that that there were significant increases in bacteria colony formation in culture plates from burn ethanol mice (11.4 × 10^3^ ± 2.05 × 10^3^) compared with sham vehicle mice (3.89 × 10^3^ ± 8.44 × 10^2^). This demonstrates that neutrophils from ethanol burn mice have reduced bacterial killing ability.

### IL-23 induces neutrophil release of IL-17 and IL-22 following ethanol intoxication combined with burn injury

**FIGURE 3.**
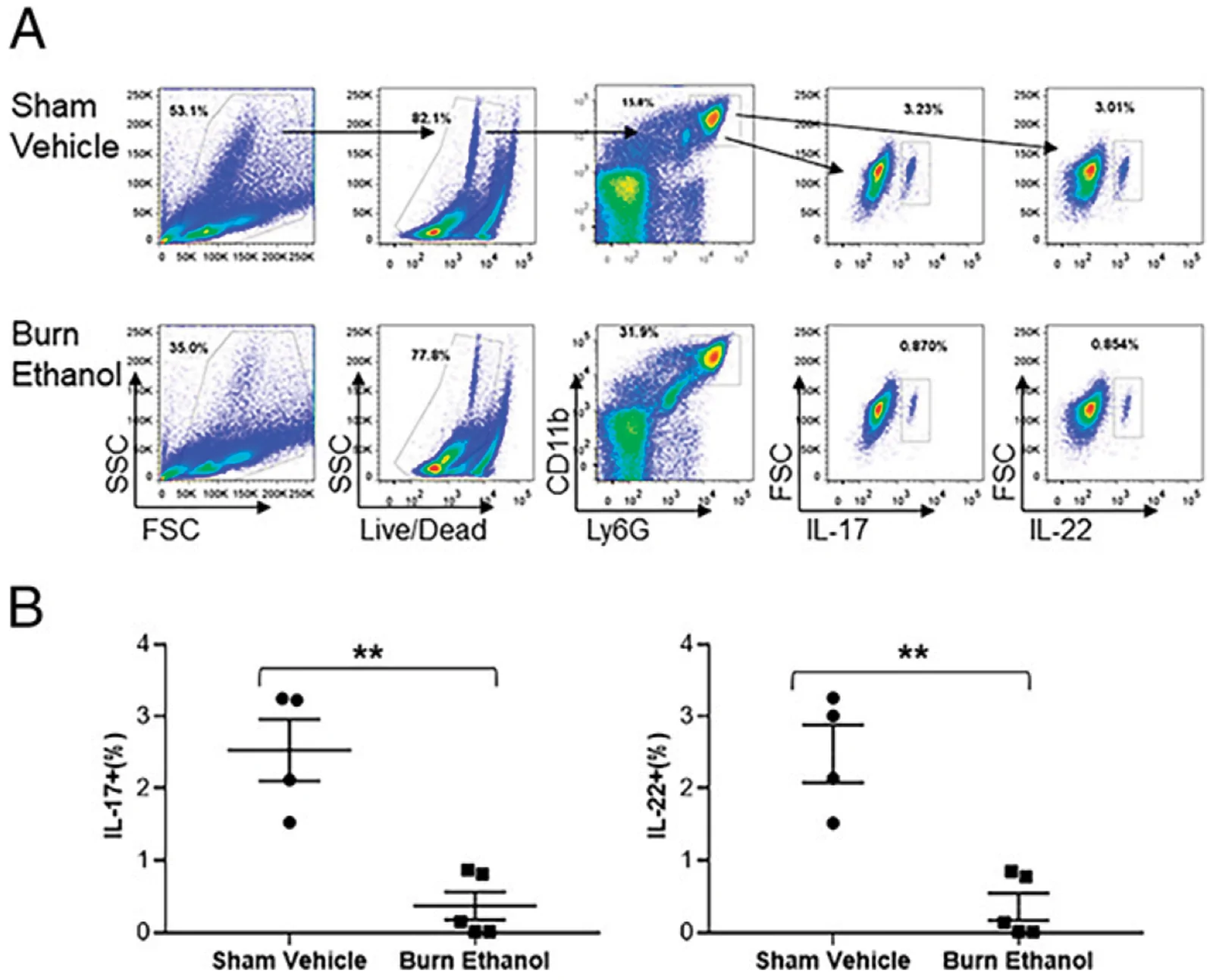
Decrease in neutrophil IL-22 and IL-17 following ethanol intoxication combined with burn injury. Blood cells (1 × 10^6^) were stimulated with Cell Stimulation Cocktail plus protein transport inhibitors for 3 h. The cells were stained with Fixable Viability Dye, CD11b−, Ly6G−, IL-17–, and IL-22–conjugated Abs. We first used forward light scatter and side scatter (of light) to gate the cells. The dead cells were then gated out as observed by Fixable Viability Dye. The live cells that expressed CD11b^+^ LY6G^+^ were considered as neutrophils. IL-17 and IL-22 expression in neutrophils by FACS (**A**). Values are means ± SEM from four to five animals per group (**B**). **p < 0.01 compared with sham vehicle group by unpaired *t* test.

**FIGURE 4.**
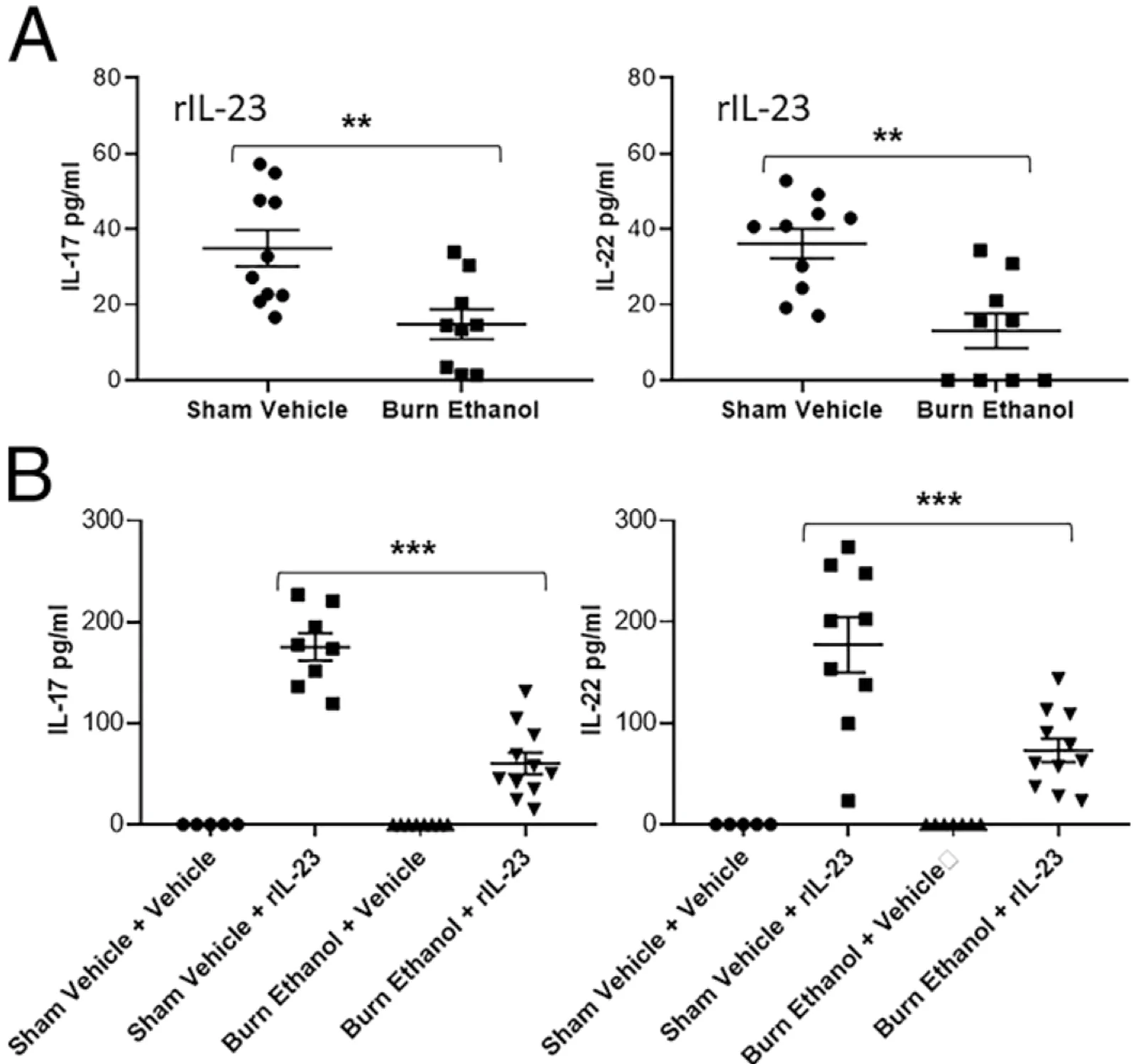
IL-23 induces neutrophil release of IL-17 and IL-22. Isolated neutrophils from blood (**A**) or peritoneal cavity (**B**) were cultured in the presence or absence of rIL-23 (10 ng/ml) for 24 h, and supernatant was collected to measure IL-17 and IL-22 level by ELISA. Values are means ± SEM from 9–11 animals per group. **p < 0.01, compared with sham vehicle group by unpaired *t* test (A) and ***p < 0.001 by one-way ANOVA Tukey multiple comparisons test (B).

Studies have indicated that Th17 effector cytokines, in particular IL-22, play a critical role for regulation of intestinal barrier following injury (17, 28). IL-17 and IL-22 are not only released by Th17 and Th22 cells but also by type 3 innate lymphoid cells, NK cells, and neutrophils. Previous studies from our laboratory have indicated that ethanol and burn injury suppressed T cell IL-17 and IL-22 release, and that IL-23 promotes T cell production of IL-17 and IL-22 (8, 29). In this study, we determined whether ethanol combined with burn injury also altered neutrophil release of IL-17 and IL-22 and whether treatment of neutrophils with rIL-23 induced IL-17 and IL-22 release following ethanol and burn injury. First, total WBCs were stimulated with Cell Stimulation Cocktail (plus protein transport inhibitors), which contains PMA, Ionomycin, brefeldin A, and monensin in cell culture medium for 3 h and stained with Ly6G-, CD11b-, IL-17–, and IL-22–conjugated Abs to analyze IL-17 and IL-22 expression in neutrophils by FACS. We observed significant decreases in expression of IL-17 and IL-22 in neutrophils harvested from burn ethanol mice compared with sham vehicle mice (Fig. 3). Second, isolated neutrophils from blood were cultured with rIL-23 (10 ng/ml) for 24 h, and supernatant was collected to measure IL-17 and IL-22 level by ELISA. As shown in Fig. 4A, there were significant decreases in IL-17 and IL-22 release by neutrophils cultured with rIL-23 in burn ethanol mice compared with sham vehicle mice. Because isolated blood neutrophils from each mouse were limited, we did not have enough neutrophils to culture in the absence of rIL-23. Finally, we isolated neutrophils from the peritoneal cavity following casein injection. We observed that IL-17 and IL-22 were undetectable in neutrophils cultured in the absence of rIL-23 from both ethanol burn and sham vehicle mice. Furthermore, neutrophils cultured in the presence of rIL-23 significantly increased release of IL-17 and IL-22 in both groups of mice. However, there were significant decreases in IL-17 and IL-22 levels in neutrophils isolated from ethanol burn mice compared with sham vehicle mice (Fig. 4B). This was a similar result observed in neutrophils isolated from blood. In the subsequent studies, we used neutrophils isolated from the peritoneal cavity because this provided the adequate cell numbers needed for our studies.

### IL-23 induces IL-23R, RORγt, Lipocalin2, and Nod2 expression in neutrophils following ethanol intoxication combined with burn injury

**FIGURE 5.**
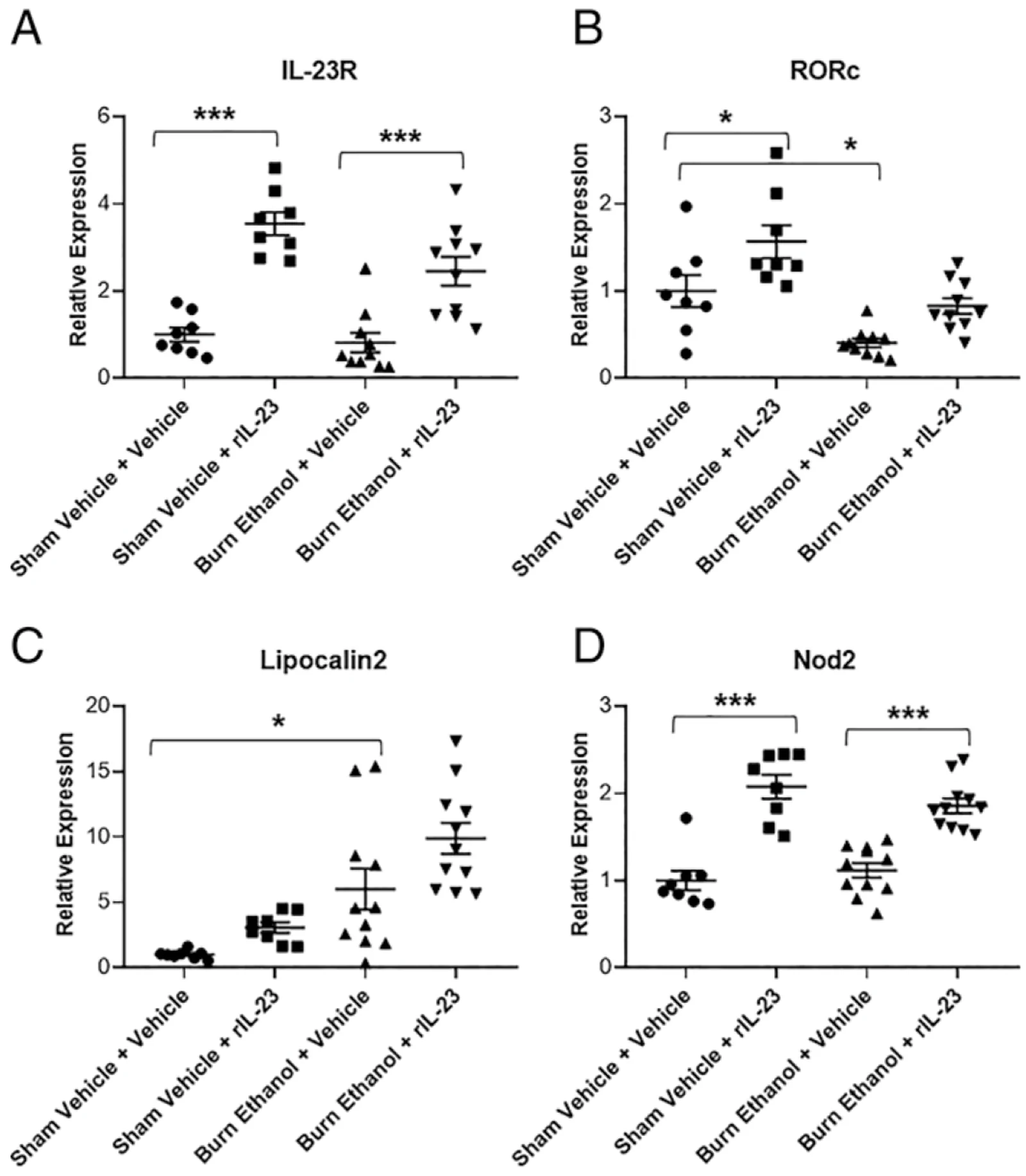
IL-23 induces IL-23R, RORγt, Lipocalin2, and Nod2 expression on neutrophils. Isolated neutrophils from the peritoneal cavity were cultured in the presence or absence of rIL-23 for 24 h, and neutrophil mRNA was isolated to determine IL-23R (**A**), RORγt (RORc) (**B**), Lipocalin2 (**C**), and Nod2 (**D**) expression in neutrophils by PCR. Values are means α SEM from 8–10 animals per group. *p < 0.05, ***p < 0.001 by one-way ANOVA Tukey multiple comparisons test.

RORγt is the master transcription factor for Th17 cell differentiation and promotes Th17 cells to release effector cytokines (30). Recent studies have found that neutrophils express IL-23R, Lipocalin2, and Nod2, which are also involved in the innate immune response against bacterial infection (23, 31, 32). In this experiment, we determined whether IL-23 modulated RORγt, IL-23R, Lipocalin2, and Nod2 mRNA expression in neutrophils following ethanol and burn injury. To test this, we cultured isolated neutrophils in the presence or absence of rIL-23 for 24 h, and neutrophil mRNA was isolated to determine IL-23R, RORγt, Lipocalin2, and Nod2 expression in neutrophils by RT-PCR. As shown in Fig. 5, there was a significant decrease in RORγt (RORc) mRNA expression in neutrophils isolated from ethanol burn mice compared with sham vehicle mice (Fig. 5B). Furthermore, Lipocalin2 mRNA expression was significantly increased in neutrophils isolated from ethanol and burn mice compared with sham vehicle mice (Fig. 5C). There were no differences in IL-23R and Nod2 mRNA expression in neutrophils isolated from ethanol burn and sham vehicle mice (Fig. 5A, 5D). However, the treatment of neutrophils with rIL-23 increased IL-23R, RORγt, Lipocalin2, and Nod2 mRNA expression in both ethanol burn and sham vehicle mice.

### IL-23 modulates formation of NETs following ethanol intoxication combined with burn injury

**FIGURE 6.**
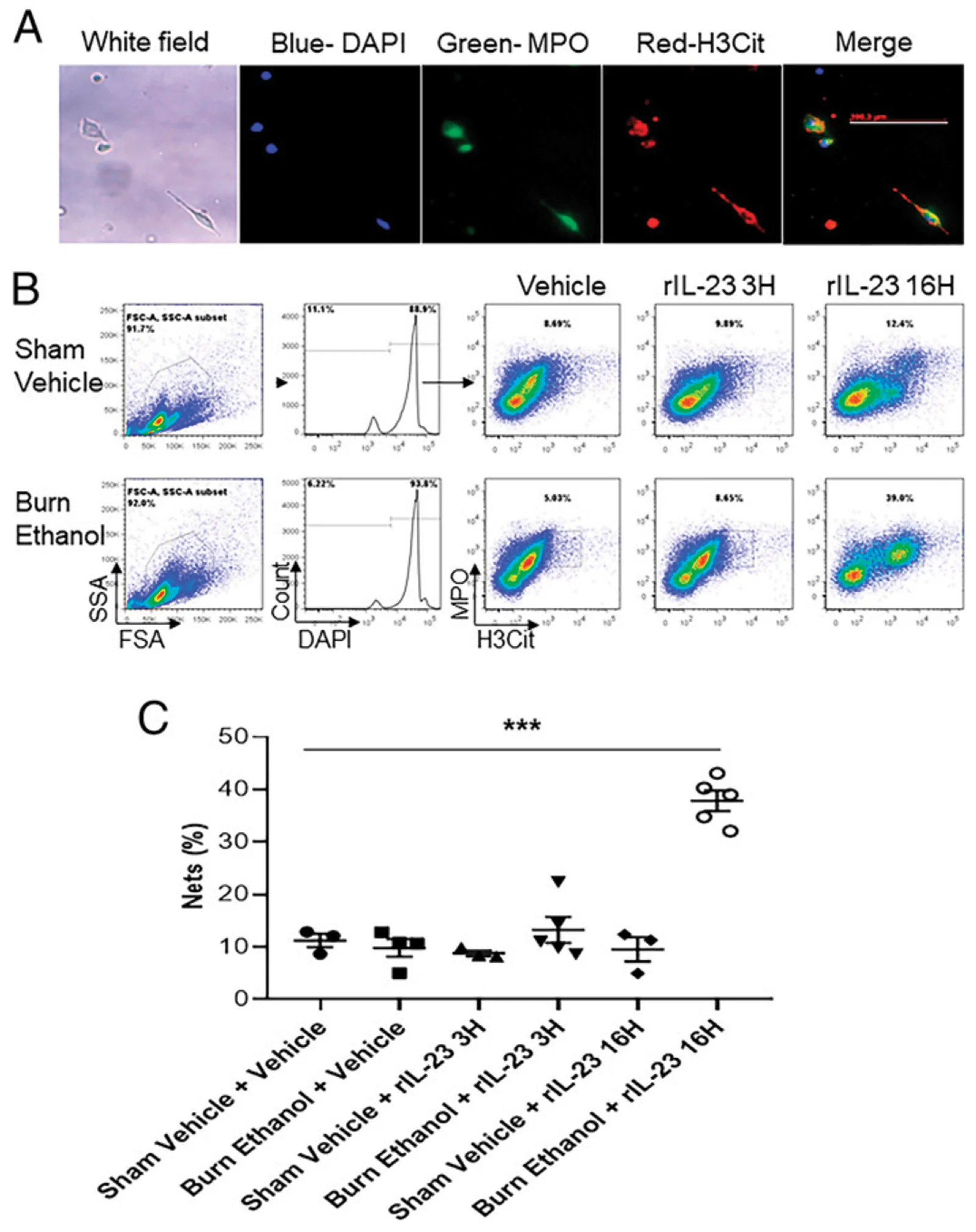
IL-23 modulates NET formation following ethanol intoxication combined with burn injury. Isolated neutrophils from the peritoneal cavity were cultured in the presence or absence of rIL-23 for 3 or 16 h. Neutrophils were fixed and stained with DAPI, H3Cit, and MPO. We first used forward light scatter and side scatter (of light) to gate the cells. The cells were then gated for DAPI^+^ cells. Double-positive cells of H3Cit and MPO were considered neutrophil NET formation by photomicrograph with ×400 original magnification (scale bar: 100.3 μM) after 16 h cultured with rIL-23 (**A**) and by FACS (**B** and **C**). Values are means ± SEM from three to five animals per group. ***p < 0.001, compared with other groups by one-way ANOVA Tukey multiple comparisons test.

Neutrophils eliminate invading pathogens through two mechanisms: phagocytosis and release of NETs. NETs are large, extracellular, web-like structures composed of decondensed chromatin associated with granular and cytosolic proteins, such as NE and MPO. These components in the NETs are proposed to trap and kill bacteria extracellularly. Histones play a critical role in chromatin architecture, and histone citrullination (H3Cit) is considered a key marker for early NET formation (27). We determined whether IL-23 regulated neutrophil NET release after ethanol and burn injury. Isolated neutrophils were cultured in the presence or absence of rIL-23 for 3 or 16 h and collected. Neutrophils were fixed and stained with DAPI, H3Cit, and MPO. H3Cit and MPO double-positive cells were considered to be NET-forming neutrophils. Neutrophil NETs were quantified by FACS (27). To confirm the structure of NETs, we visualized the cells by microscopy (Fig. 6A). We observed that there were no differences in neutrophil NET formation from neutrophils cultured in the presence or absence of rIL-23 for 3 h. However, a significant increase in neutrophil NET formation was observed in neutrophils cultured with rIL-23 for 16 h in the burn ethanol mice (Fig. 6).

### IL-23 modulates neutrophil bacterial killing following ethanol intoxication combined with burn injury

**FIGURE 7.**
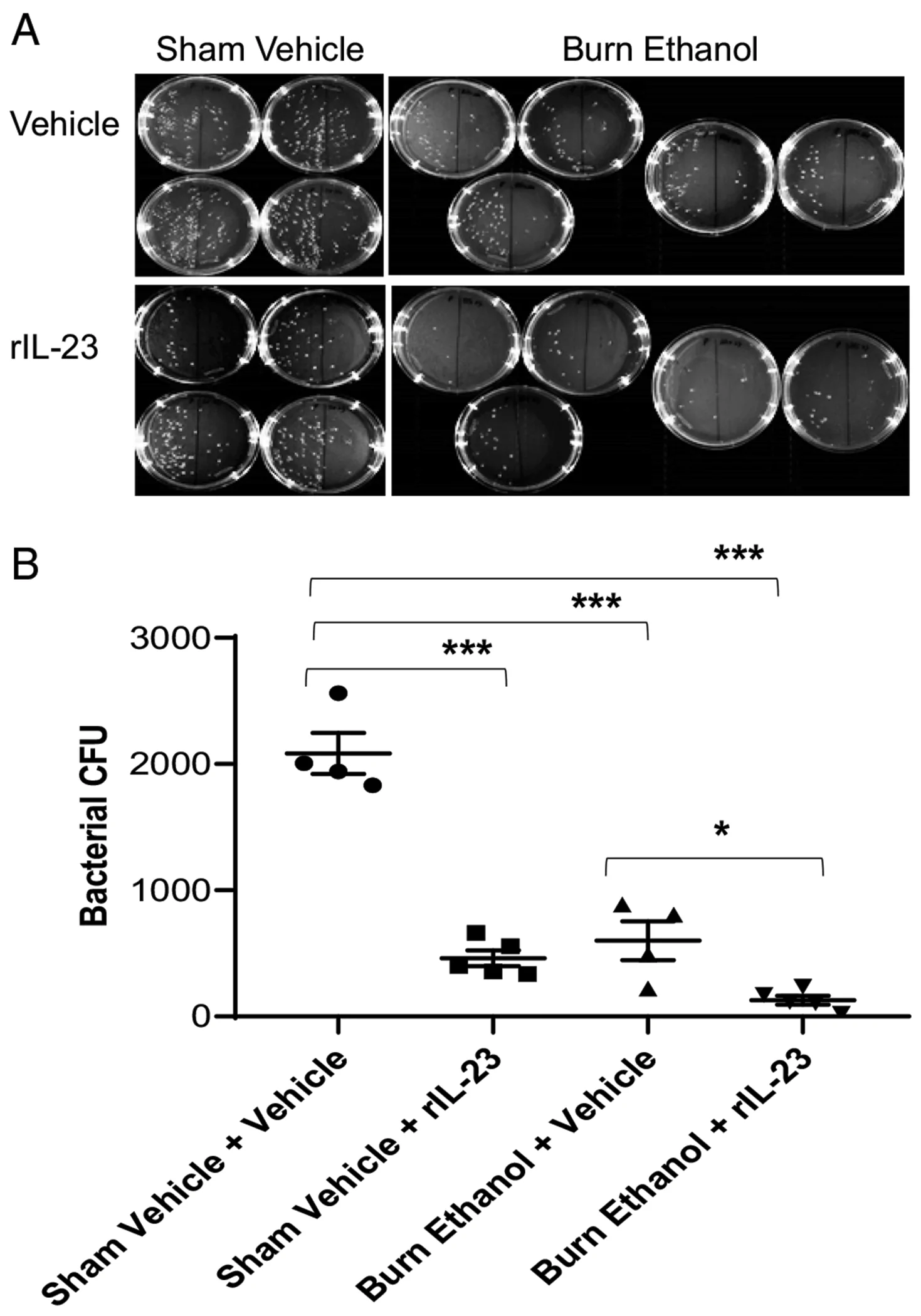
IL-23 enhances neutrophil ability of bacterial killing. Isolated neutrophils (3 × 10^5^) from the peritoneal cavity were cultured in the presence or absence of rIL-23 for 16 h. Neutrophils were washed and incubated with 9 × 10^6^ CFUs *E. coli* (1:30) for 1 h. The extracellular bacteria were killed by using gentamicin, penicillin, and streptomycin. The neutrophils were lysed with H_2_O. The 10 μl of cell lysis was placed on the tryptic soy agar plate and incubated at 37°C overnight. The bacteria colonies were counted and calculated for each sample. Each plate contained two concentrations of cell lysate. The left half of the plate shows undiluted cell lysates, and the right half shows a 10× dilution of cell lysate (**A**). Values are means ± SEM from four to five animals per group (**B**). *p < 0.05, ***p < 0.001 by one-way ANOVA Tukey multiple comparisons test.

Because IL-23 induces neutrophil release of NETs, we determined whether IL-23 contributed to bacterial killing. Isolated peritoneal neutrophils were cultured with rIL-23 for 16 h, and bacterial killing was determined as described previously. As shown in Fig. 7, we observed that there was a significant increase in bacterial colonies from neutrophils isolated from sham vehicle mice compared with ethanol burn mice. This was in contrast to our previous experiment (Fig. 2), in which we isolated neutrophils from peripheral blood. Blood neutrophils are mature and function as the first line of defense to destroy pathogenic organisms. Unfortunately, isolation of neutrophils from mouse blood resulted in insufficient numbers for many of our experiments; less than ~1 ml of blood is collected from each mouse and results in <1 million isolated neutrophils. Therefore, we isolated neutrophils from the peritoneal cavity by injection of casein. However, treatment of neutrophils with rIL-23 significantly decreased bacterial colonies in neutrophils from both sham vehicle mice and burn ethanol mice (Fig. 7).

**FIGURE 8.**
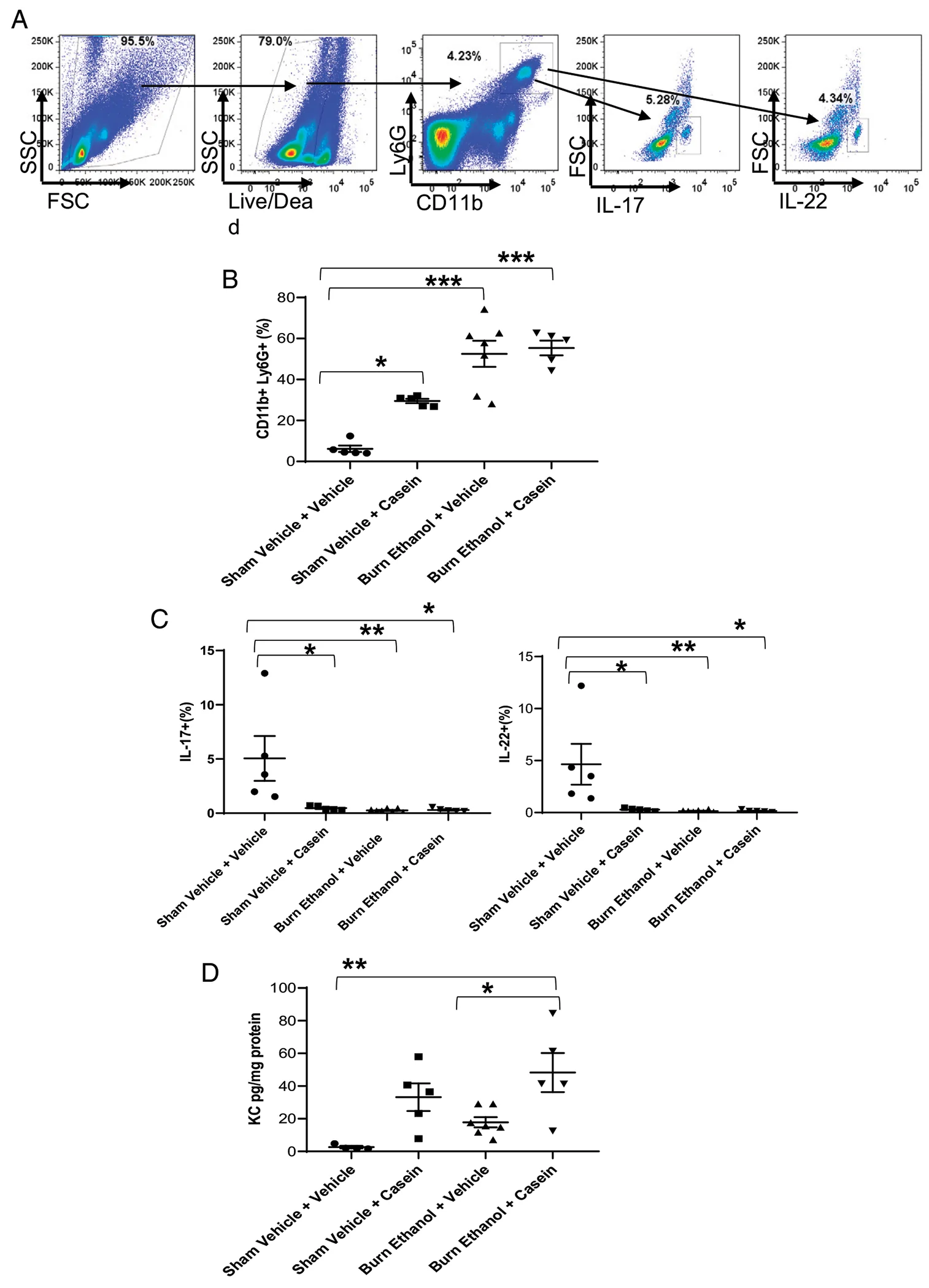
Injection of casein i.p. alters neutrophil function. After burn injury, mice received twice i.p. injection of casein. One day after injury, mice were euthanized. The peripheral blood and small intestine were collected. The RBCs were lysed. Blood cells (1 × 10^6^) were stimulated with Cell Stimulation Cocktail plus protein transport inhibitors for 3 h. The cells were stained with Fixable Viability Dye, CD11b, Ly6G, IL-17, and IL-22. We first used forward light scatter and side scatter (of light) to gate the cells. The dead cells were then gated out as observed by Fixable Viability Dye. The live cells that expressed CD11b^+^ LY6G^+^ were considered as neutrophils. IL-17 and IL-22 expression in neutrophils by FACS (**A–C**). The small intestine was homogenized to determine KC levels and nominalized by protein (**D**). Values are means ± SEM from five to seven animals per group. *p < 0.05, **p < 0.01, ***p < 0.001 by one-way ANOVA Tukey multiple comparisons test.

**FIGURE 9.**
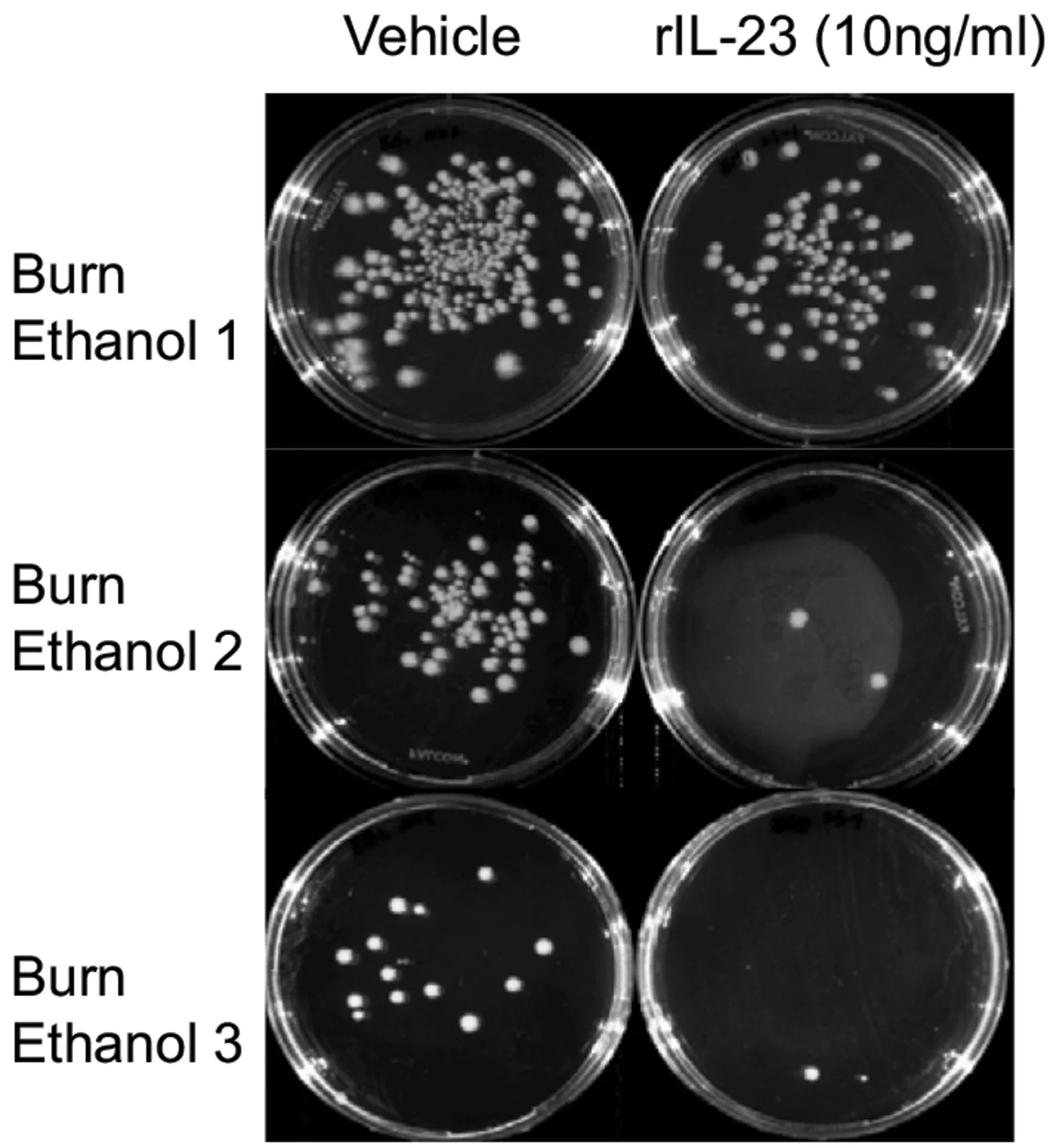
IL-23 modulates bacterial killing in blood neutrophils. Isolated blood neutrophils (3 × 10^5^) from three burn ethanol mice were cultured in the presence or absence of rIL-23 for 16 h. Neutrophils were washed and incubated with 9 × 10^6^ CFUs *E. coli* (1:30) for 1 h. The extracellular bacteria were killed by using gentamicin, penicillin, and streptomycin. The neutrophils were lysed with H_2_O. The 10 μl of cell lysate was placed on the tryptic soy agar plate and incubated at 37°C overnight.

Several studies suggested that blood neutrophils and exudate neutrophils are functionally different (33, 34). To confirm this, we injected mice i.p. with casein, and blood and small intestine tissue were collected. The RBCs were lysed and white cells were stained with conjugated Ly6G, CD11b, IL-17, and IL-22 Abs. The expression of IL-17 and IL-22 in neutrophils was determined by FACS. We observed that sham vehicle mice injected with casein have an increased neutrophil population in the blood (Fig. 8A, 8B), which was accompanied with a decrease in blood neutrophil IL-17 and IL-22 expression (Fig. 8C) compared with sham vehicle mice injected with vehicle. There were no differences in neutrophil population in blood or expression of IL-17 and IL-22 in blood neutrophils from burn ethanol mice regardless of injection of casein. In addition, we observed that there was a significant increase in keratinocyte-derived chemokine (KC) production from small intestinal tissue of sham vehicle mice injected with casein compared with sham vehicle mice injected with vehicle (Fig. 8D). There was an increased trend in KC levels in small intestinal tissue from burn ethanol mice injected with casein compared with burn ethanol mice injected with vehicle. Furthermore, we determined whether rIL-23 promoted bacterial killing ability in blood neutrophils after ethanol and burn injury. We observed that blood neutrophils cultured with rIL-23 decreased the number of colonies on the culture plate compared with vehicle-cultured neutrophils after ethanol and burn injury (Fig. 9).

## DISCUSSION

In previous studies from our laboratory, we have observed that ethanol intoxication combined with burn injury increases intestinal bacterial growth, disrupts the intestinal barrier, and enhances bacterial translocation (9, 10, 35). During intestinal damage or bacterial invasion, neutrophils rapidly extravasate from blood vessels to migrate to inflammatory sites. Neutrophils are activated by microbial/bacterial products (LPS and peptidoglycan) and inflammatory cytokines (IL-8, TNF-α, and IL-1β), and destroy pathogens using several strategies. First, the activation of neutrophils leads to reduction of superoxide by the enzyme NADPH oxidase. Superoxide is converted to other ROS, such as superoxide radical (O_2_^−^) and hydrogen peroxide, that are effective at killing bacteria. Second, neutrophils directly kill bacteria by phagocytosis. Activated neutrophils express endocytic pattern recognition receptors that recognize and bind to microbial pathogen-associated molecular patterns. Neutrophils engulf the microbe to form a phagosome, which then fuses with the lysosome containing various digestive enzymes, antimicrobial peptides, and proteins to kill bacteria intracellularly. Finally, neutrophils can also kill pathogens extracellularly by releasing NETs. These are large, extracellular, web-like structures composed of chromatin and neutrophil granular proteins. Neutrophils stimulated with PMA or specific agonists result in the activation of NADPH oxidase and ROS generation. Afterward, protein-arginine deiminase 4 is activated and citrullinates arginine residues on histones in neutrophils to cause chromatin decondensation. MPO and NE translocate from cytoplasmic azurophilic granules to the nucleus and further the decondensation of chromatin. Subsequently, the nuclear membrane ruptures and chromatin is released into the cytosol to mix with granule contents. Finally, the preformed NETs are released into the extracellular space. NETs entrap bacteria and kill them by high local concentrations of antimicrobial components extracellularly. However, excess activated neutrophils can also cause tissue damage in various inflammatory conditions, such as trauma, burn, and sepsis (36, 37). Consistent with these studies, we have shown neutrophil-mediated intestine and lung tissue damage in a rat model of ethanol and burn injury (38–40).

We observed that there was a significant decrease in phagocytosis and bacterial killing ability in blood neutrophils isolated from ethanol burn injured mice compared with sham vehicle mice, and this may result in bacterial growth in ethanol burn mice. Studies have reported that IL-17 and IL-22 regulate the intestinal barrier, including maintenance of the gut epithelial lining and induction of antimicrobial peptides to prevent microbial invasion (23, 24, 41). Recent studies from our laboratory have indicated that ethanol intoxication combined with burn injury globally suppresses T cell IL-17 and IL-22 release, which may contribute to decrease host defense and increased susceptibility to infection after alcohol and burn injury. IL-23 is released by APCs (e.g., dendritic cells), binds to its receptor, and activates the JAK/STAT pathway. The phosphorylation of STAT3 leads to activation of RORγt and AHR transcription factors, which are involved in the release of IL-22 and IL-17 in T cells. A recent study has indicated that neutrophil granules contain IL-22 and that neutrophils are also an important source of IL-22. We determined whether ethanol intoxication combined with burn injury influences IL-22 and IL-17 release in neutrophils. We observed that there was a significant decrease in IL-22 and IL-17 in blood neutrophils isolated from burn ethanol mice compared with sham vehicle mice. Because isolated blood neutrophils from each mouse were limited, we isolated neutrophils from the peritoneal cavity in response to casein injection for further experiments. We observed that IL-17 and IL-22 were undetectable in neutrophils isolated from peritoneal neutrophils from both ethanol burn mice and sham vehicle mice. However, when cultured with rIL-23, there were significant increases in the release of IL-17 and IL-22 in both groups of mice. Nevertheless, similar to the result observed in blood neutrophils, the release of IL-17 and IL-22 by peritoneal neutrophils remained significantly lower in ethanol burn mice compared with sham vehicle mice. We also observed that IL-23 promoted expression of IL-23R and RORγt, but not AHR (data not shown). Consistent with this finding, recent studies have indicated that IL-23 is required for promoting neutrophil IL-22 expression in DSS-induced colitis. IL-22 levels in colonic tissue were significantly decreased in IL-23–deficient mice compared with wild-type mice (24). Inhibition of RORγt abolished IL-23–induced IL-17 and IL-22 production in neutrophils (23). In addition, we observed that IL-23 promotes expression of Lipocalin2, an antimicrobial peptide, and Nod2. Furthermore, we determined whether IL-22– and IL-17–producing neutrophils contribute to NET formation and bacteria killing. We used flow cytometric assays for direct quantification of neutrophil NET formation (27). There were no differences in NET formation in unstimulated neutrophils between sham vehicle mice and burn ethanol mice. Although not significant, there was an increased trend of NET formation in neutrophils treated with rIL-23 for 3 h in burn ethanol mice compared with sham vehicle mice. However, in neutrophils treated with rIL-23 for 16 h, there was a significant increase in NET formation in burn ethanol mice compared with sham vehicle mice. We also observed that neutrophils treated with rIL-23 for 16 h had significant increases in bacteria killing compared with neutrophils treated with vehicle in both sham vehicle and burn ethanol mice. However, we found that neutrophils treated with vehicle had significantly increased bacterial killing ability in burn ethanol mice compared with sham vehicle mice. This is in contrast to neutrophils isolated from peripheral blood (Fig. 2), where we observed a decrease in bacterial killing. Due to poor yield of neutrophils from peripheral blood (<1× 10^6^ cells per mouse), most studies use neutrophils isolated from the peritoneal cavity or bone marrow to obtain sufficient numbers. Isolated cells from the bone marrow contain immature neutrophils. Under normal conditions, the peritoneal cavity does not contain many neutrophils. To isolate neutrophils from the peritoneal cavity, we used an i.p. injection of an agent, such as thioglycollate or casein, to induce sterile inflammation and recruit neutrophils to the injection site. These neutrophils exhibited an activated phenotype. Ganer et al. (42) reported that peritoneal cavity neutrophils extracted by sodium caseinate enhance killing of *B. dermatitidis* compared with blood neutrophils. However, thioglycollate medium–extracted neutrophils have decreased ability to kill *B. dermatitidis* compared with blood neutrophils (34, 42). The mechanism for the differential killing ability is still unclear. We observed that there was a significant increase in blood neutrophil population and decreased expression of IL-17 and IL-22 in neutrophils from sham vehicle mice injected i.p. with casein compared with mice injected with vehicle. We also observed that uninjured mice injected i.p. with casein had increased KC production in small intestinal tissue compared with those injected with vehicle. This is indicative that mice injected with casein mount an inflammatory response, including neutrophil infiltration. Neutrophils isolated from the peritoneal cavity treated with rIL-23 had enhanced bacterial killing ability in both sham vehicle and burn ethanol mice. To confirm whether rIL-23 also regulates blood neutrophil bacteria killing, we treated blood neutrophils with rIL-23. The results demonstrate that blood neutrophils cultured with rIL-23 also increased bacterial killing in mice receiving ethanol and burn injury (Fig. 9).

In some of our experimental analyses, neutrophils were cultured for overnight (i.e., 24 h), and concerns can be raised regarding their viability because blood neutrophil lifespan is normally considered to be <24 h (half-life 6–8 h). However, the lifetime of mammalian neutrophils appears to be controversial. For example, inflammation can alter neutrophil lifespan. A previous study from our laboratory has shown that ethanol combined with burn injury decreased neutrophil caspase-3 activity and apoptosis (39). Pillay et al. (43) indicated that an average human circulatory neutrophil lifespan was 5.4 d, and the half-life of circulating neutrophils in mice was 9–18 h (43, 44), although in this study, we did not determine the neutrophil lifespan. We cultured an equal number of neutrophils from various experimental conditions and compared the neutrophil-producing IL-17 and IL-22 in burn ethanol mice with shams. However, neutrophils cultured with rIL-23 for 24 h produced high levels of IL-17 and IL-22, specifically in peritoneal cavity neutrophils isolated from sham vehicle animals. We used 3-h stimulation and overnight culture, and both show a decrease in IL-17/22.

In conclusion, our findings indicate that ethanol intoxication combined with burn injury suppresses neutrophil ability of phagocytosis and bacteria killing, which is accompanied by decreased neutrophil release of IL-22 and IL-17. The treatment of neutrophils with IL-23 significantly increases neutrophil IL-22 and IL-17 release and promotes expression of IL-23R, RORγt, Lipocalin2, and Nod2 following ethanol and burn injury. Furthermore, IL-22– and IL-17–producing neutrophils have enhanced NET formation and bacterial killing ability, which is dependent on IL-23. Our studies suggested that IL-23 induction of IL-22 and IL-17 production in neutrophils may play a critical role in protection against bacterial infection following ethanol and burn injury. These findings may help in developing the new therapeutic strategies for ethanol and burn injury patients.

## Abbreviations used in this article:

KC: keratinocyte-derived chemokine
MPO: myeloperoxidase
NE: neutrophil elastase
NET: neutrophil extracellular trap
Nod2: Nod-like receptor 2
RORγt: retinoic acid–related orphan receptor γt
ROS: reactive oxygen species
